# Catalyst-Free Synthesis of Lignin Vitrimers with Tunable Mechanical Properties: Circular Polymers and Recoverable Adhesives

**DOI:** 10.1021/acsami.1c17412

**Published:** 2021-11-23

**Authors:** Adrian Moreno, Mohammad Morsali, Mika H. Sipponen

**Affiliations:** Department of Materials and Environmental Chemistry, Stockholm University, Svante Arrhenius Väg 16C, SE-106 91 Stockholm, Sweden

**Keywords:** lignin, biobased vitrimers, recyclable adhesives, organic polymers, one-pot synthesis

## Abstract

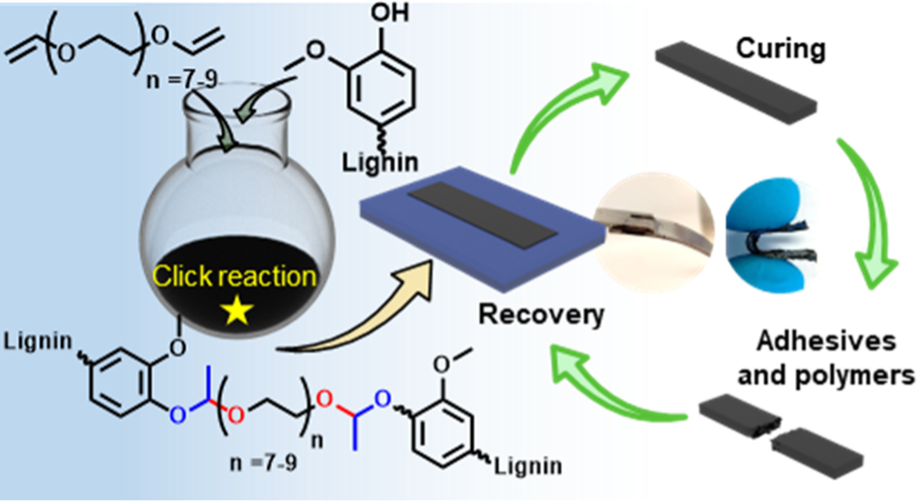

Biobased circular materials are alternatives to fossil-based engineering plastics, but simple and material-efficient synthetic routes are needed for industrial scalability. Here, a series of lignin-based vitrimers built on dynamic acetal covalent networks with a gel content exceeding 95% were successfully prepared in a one-pot, thermally activated, and catalyst-free “click” addition of softwood kraft lignin (SKL) to poly(ethylene glycol) divinyl ether (PDV). The variation of the content of lignin from 28 to 50 wt % was used to demonstrate that the mechanical properties of the vitrimers can be widely tuned in a facile way. The lowest lignin content (28 wt %) showed a tensile strength of 3.3 MPa with 35% elongation at break, while the corresponding values were 50.9 MPa and 1.0% for the vitrimer containing 50 wt % of lignin. These lignin-based vitrimers also exhibited excellent performance as recoverable adhesives for different substrates such as aluminum and wood, with a lap shear test strength of 6.0 and 2.6 MPa, respectively. In addition, recyclability of the vitrimer adhesives showed preservation of the adhesion performance exceeding 90%, indicating a promising potential for their use in sustainable circular materials.

## Introduction

Cross-linked polymers, also known as thermosets, are one of the most important classes of polymeric materials owing to their permanent covalent bond structure, which renders them with outstanding mechanical properties and resistance to solvents and heat, both of which are of broad importance in industrial applications.^1^ However, thermosets are often made from fossil raw materials and suffer from a lack of appropriate repairing or recycling methods. Consequently, most of them are disposed of in landfills or incinerated.^2^ The increasing attention to circular economy and legislation to phase out single-use plastics are also driving the transformation of the polymer industry toward biobased and recyclable materials. In this context, dynamic polymers have been developed by the introduction of labile non-covalent^3,4^ and dynamic covalent^5−11^ bonds capable of undergoing reversible formation, which endow these materials with improved properties and more importantly creates the possibility to recycle them.

This dynamic bond formation concept was first applied to thermoset materials by Leibler and co-workers in 2011,^12^ showing that covalent bonds present in a cross-linked polymeric structure could be rendered reversible in the presence of a zinc acetate catalyst. These new types of materials were coined as vitrimers, and after that, a variety of materials with dynamic covalent bond exchange reactions have been developed based on transesterification,^13^ boronic ester exchange,^14,15^ transalkylation,^16^ olefin metathesis,^17^ imine chemistry,^18,19^ Diels–Alder reactions,^20^ vinylogous urethane/urea transamination,^21−23^ and so on. From particular interest, it has recently been reported that dynamic covalent networks based on acetal exchange reactions are robust,catalyst-free and hold the potential to recycle polymers in a simple way.^24,25^ However, most of the vitrimers described in the literature contain synthetic polymers derived from fossil resources.^26−30^ This fact, together with the concerns about rising plastic pollution and greenhouse gas emissions, has brought renewable polymers into the equation.^31−33^

Natural polymers such as lignin have distinct chemical structures and unique properties that make them attractive and potentially sustainable components of vitrimers. Following this trend, last years have experienced coherent development of biobased vitrimers, owing to the lucrative properties of vitrimers and the relatively high abundance and low cost of the raw materials.^13,34−44^ However, in most cases, chemical modification of the bio-based starting materials is needed to generate synthons able to react and form dynamic covalent networks, in addition to the use of catalysts to trigger the reversibility of dynamic covalent bonds.^13,37,40−42^ This pre-processing or fractionation of natural polymers, together with the need of catalysts, hinders their scalability toward industrial processes and therefore limits their potential applications. This present scenario poses an important challenge to avoid the chemical or physical pre-modification and functionalization of renewable raw materials that are available in a sufficient scale and at a low cost for industrial end uses.

In this context, lignin as the most abundant aromatic natural polymer and industrial byproduct appears as an excellent resource for the production of value-added products, as has been demonstrated during the last years.^45−53^ However, most of the lignin-based polymers so far developed do not qualify as circular materials. There are only a few published studies of lignin-based reprocessable polymers, including thermally or catalytically cleavable ester linkages between chemically modified or fractionated kraft lignin with sebacic acid derivatives,^13^ poly(ethylene glycol) (PEG),^54^ or PEG-epoxy cross-linkers.^55^ Another example is the use of etherified enzymatic hydrolysis lignin with bisphenol A-based epoxy cross-linkers to produce thermally cleavable ester-linked vitrimers.^56^ These materials contain up to 40–67 wt % of lignin but require processing or fractionation of lignin with multiple synthetic steps and often a catalyst to trigger the covalent bond exchange, which is not ideal from environmental or economical points of view.

**Figure 1:**
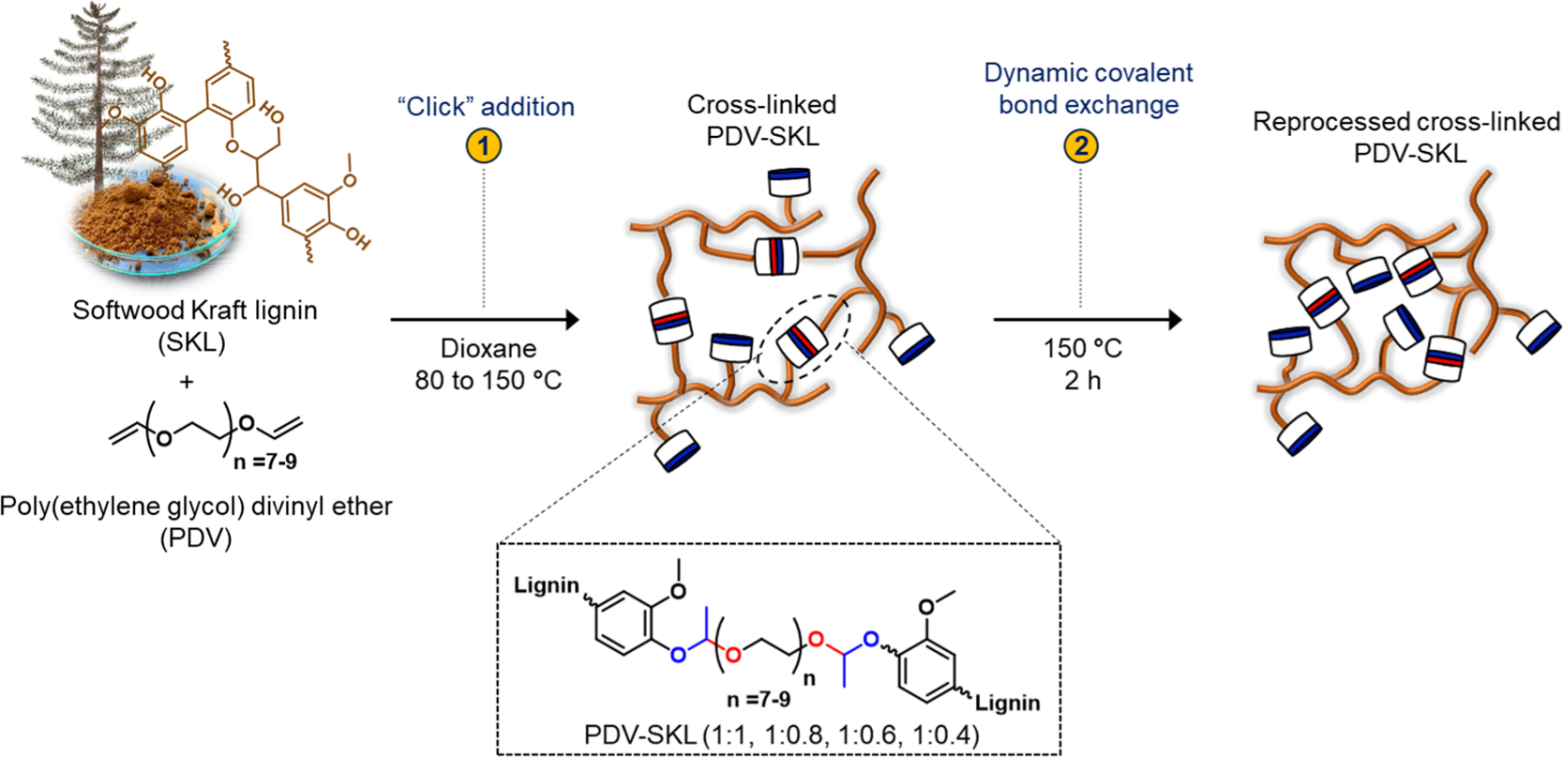
Schematic preparation of lignin-based vitrimers (PDV-SKL) and its recovery through catalyst-free dynamic acetal exchange reactions.

Here, relying on acetal exchange reactions,^24,25^ we report for the first time a one-pot catalyst-free preparation of lignin-based vitrimers. Our approach exploits the aliphatic and phenolic hydroxyl groups of softwood kraft lignin (SKL) to conduct the thermally catalyzed addition reaction with a commercially available poly(ethylene glycol) divinyl ether (PDV), resulting in a dynamic network based on thermally labile acetal linkages (Figure 1). Our work avoids the chemical functionalization or fractionation of lignin, which are common methods to increase reactivity and overcome the poor miscibility of lignin in polymeric matrices, and instead offers a direct route from lignin to vitrimers in mass recovery yields exceeding 95%. We also demonstrate that the mechanical properties of the lignin-based vitrimers can be tuned simply by varying the weight fraction of lignin in the vitrimer. Last but not least, we demonstrate the lignin-based vitrimers as recoverable adhesives for metal and wood substrates, with preservation of adhesive strength exceeding that of benchmark materials.

## Results and Discussion

Our approach to lignin-based vitrimers starts with the preparation of the lignin-based acetal dynamic networks through a thermal, catalyst-free “click” addition of SKL to poly(ethylene glycol) divinyl ether (PDV) (Figure 1). The traditional way of acetal formation by this route involves the use of strong acids (*e.g.*, pyridinium *p*-toluenesulfonate) as a catalyst under mild reaction conditions (*e.g.*, room temperature).^57−59^ However, we decided to avoid acid catalysis to simplify our approach and avoid possible acid traces remaining after the synthesis that could catalyze the degradation of acetals, severely affecting the stability of the covalent cross-linking network. In addition, the thermal “click” addition of hydroxyl groups with divinyl ethers has previously been studied with small-molecule compounds, showing excellent yields (>95%) and also proving that the dynamic exchange mechanism is a combination of acetal metathesis and transacetalization.^24,25^ The selection of SKL as a starting material was rationalized based on the inherent rigidity and presence of multiple aliphatic and phenolic hydroxyl groups, which are expected to provide enough cross-linking sites for the formation of dynamic covalent networks.^54^ As a soft segment, we chose PDV that contains a flexible ether chain that can alleviate the stiffness arising from lignin. In addition, PDV acts as a co-solvent due to its ability to dissolve SKL and therefore allows reduction of the amount of organic solvent (dioxane, 40% by volume in the liquid phase), which would be beneficial for industrial applications and reduce the environmental impact of the synthesis of this type of materials.

Small model compounds were used to investigate and prove the kinetics of the acetal exchange reactions. First, owing to the presence of carboxylic groups in SKL, the possibility that these groups could act as a co-catalyst and promote the reaction between hydroxyl groups and divinyl ethers was studied. For this purpose, guaiacol was reacted *via* a thermally induced “click” reaction with butyl vinyl ether in the presence or absence of acetic acid as a homogenous catalyst and mimicking the same molar ratio present in SKL (molar ratio of carboxylic acid to the sum of aliphatic and phenolic OH groups = 0.057) (Figures S1 and S3). Kinetic experiments showed no significant differences (*k* = 0.0150 min^–1^ without acetic acid and *k* = 0.0098 min^–1^ with acetic acid), indicating that carboxylic acid in this range of concentration would not act as a co-catalyst (Figure S1). The postulated mechanism behind the acetal exchange reactions (transacetalization and acetal metathesis) was also evaluated. The product derived from the coupling between guaiacol and butyl vinyl ether was further reacted with isopropanol at 80 °C and monitored by ^1^H NMR spectroscopy. ^1^H NMR spectra of the reaction mixture after 5 h showed the presence of new acetal peaks in the region of 4.5–5.6 ppm, which is indicative of the thermally induced and catalyst-free dynamic acetal exchange reactions, following the mechanisms of acetal metathesis and transacetalization, as previously reported (Figure S2).^24,25^

**Figure 2:**
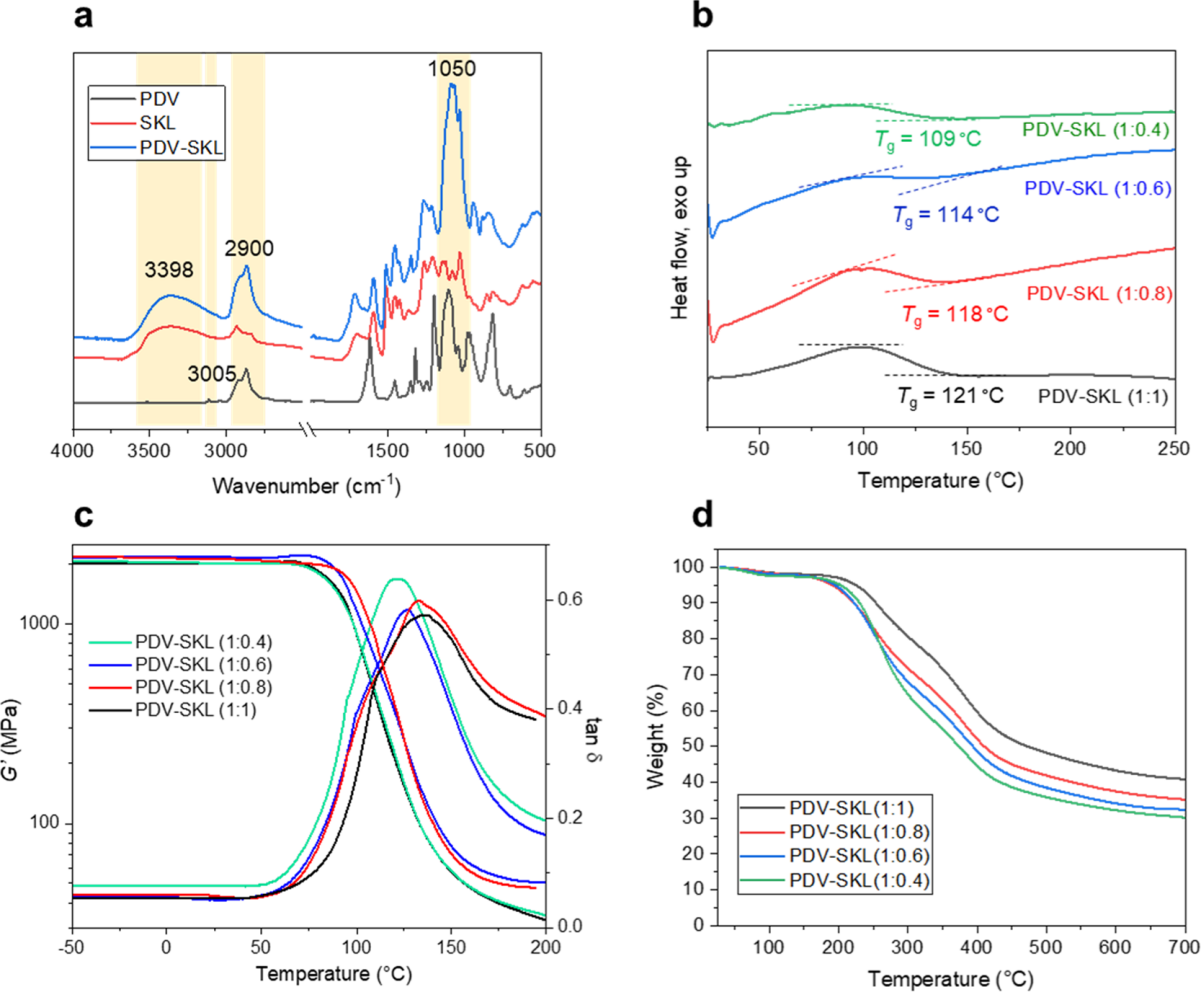
Characterization of lignin-based vitrimers (PDV-SKL): (a) FT-IR spectra of SKL, PDV, and PDV-SKL (1:1). (b) DSC thermograms, (c) storage modulus and tan δ, and (d) TGA thermograms for PDV-SKL vitrimers with different SKL contents.

Inspired by the rapid acetal exchange observed in the model study, four lignin-based vitrimers (PDV-SKL) with different contents of SKL (from 28 to 50 wt %) were prepared to evaluate their different properties (Table 1). In all cases, a gel content higher than 95% was determined by extraction with a mixture of tetrahydrofuran/dioxane (3:1, v/v), indicating an extensive formation of the cross-linking networks during the synthetic step. The successful cross-linking process and the change in functional groups before and after the reaction between SKL and PDV at various mass ratios were monitored by FTIR spectroscopy. As a representative example, the spectra of SKL, PDV, and the PDV-SKL (1:1) vitrimer after the curing process are shown in Figure 2a. The clear decrease in the intensities of the stretching bands corresponding to the double bond (=C–H, 3005 cm^–1^, and C=C, 1460 cm^–1^) in PDV (Figures 2a and S4), together with the increased intensities of the stretching bands corresponding to the alkyl chain (2900 cm^–1^) and carbon–oxygen bond (acetal) (1050 cm^–1^) in PDV-SKL (1:1) in comparison to the starting materials (SKL and PDV), supports unequivocally the formation of the acetal dynamic covalent networks.

**Table 1:** Composition of PDV-SKL Vitrimers and Their Thermal and Mechanical Properties

							mechanical properties		
sample composition	SKL (wt %)^a^	gel content (%)^b^	*T*_g_ (°C)^c^	*T*_d5%_ (°C)^d^	*T*_d30%_ (°C)^e^	*T*_s_ (°C)^f^	modulus (MPa)	tensile strength (MPa)	elongation (%)
PDV-SKL (1:1)	50	98	121	224	357	149	2100	50.9	1.0
PDV-SKL (1:0.8)	44	96	118	191	310	150	780	33.9	8.1
PDV-SKL (1:0.6)	37	97	114	195	291	124	320	15.0	15.1
PDV-SKL (1:0.4)	28	95	109	202	280	122	12.6	3.3	35.0

a Weight percent of SKL in the vitrimer material.

b The gel content was measured by extraction with the mixture of tetrahydrofuran/dioxane (3:1, v/v).

c From DSC curve (Figure 2c).

d Temperature corresponding to 5 wt % mass loss.

e Temperature corresponding to 30 wt % mass loss.

f Static heat resistance temperature calculated from eq 1.

The stretching band at 3400 cm^–1^ corresponds to the hydroxyl groups of SKL. Its intensity decreased only slightly due to the curing process, indicating that at 1:1 weight ratio of SKL to PDV, hydroxyl groups remain available, which in turn ensures complete reactivity of the divinyl ether moieties of PDV. The excess of hydroxyl groups promotes faster exchangeability of dynamic acetal bonds *via* the transacetalization pathway as shown in the small model compound experiments (Figure S2). Quantitative ^31^P NMR spectroscopy of initial SKL also supports the presence of an excess of hydroxyl groups relative to the divinyl ether moieties (Figure S3 and Table S1).^60^ The non-isothermal DSC curve of the mixture of PDV and SKL also exhibited a strong exothermic peak between 100 and 200 °C (Figure S5), which indicates that the curing process occurred thermally, and is the reason for our choice of 150 °C as the curing temperature. The DSC thermograms of PDV-SKL vitrimers shown in Figure 2b show that the glass transition temperatures (*T*_g_) of the vitrimers systematically increased with increasing lignin content, as reported previously.^13,48^ This fact is interrelated with the cross-linking density and the rigidity of the covalently cross-linked chains that increase in abundance with the lignin content due to its inherently complex and rigid structure with multiple hydroxyl groups as cross-linking points. The *T*_g_ was also determined from the peak temperature of tan δ (Figure 2c). The peak maxima of tan δ at 140, 135, 130, and 121 °C were assigned as the *T*_g_ for PDV–SKL (1:1), (1:0.8), (1:0.6), and (1:0.4), respectively. The *T*_g_ trend was consistent with the one observed by DSC, which further indicates that the rigid structure of lignin and high cross-linking density led to the increase in *T*_g_. The thermal stability of PDV-SKL vitrimers, which is critical for their final applications (*e.g.*, in coatings or adhesives), was investigated by thermogravimetric analysis (TGA) in a nitrogen atmosphere, revealing two major weight loss stages (Figure 2d). The first one is situated between 210 and 300 °C for all the compositions and is associated to the cleavage of lignin linkages,^61^ while the second one situated between 300 and 400 °C is associated to the thermal decomposition of the acetal linkages.^57^ The initial decomposition temperature for 5% weight loss (*T*_d5%_) and the temperature for 30% weight loss (*T*_d30%_) (Table 1) were also used to calculate the static heat resistance index (*T*s) (eq 1)^24^ and evaluate the thermal stability of PDV-SKL vitrimers. PDV-SKL vitrimers showed an enhanced thermal stability as the lignin content increased, as can be observed from the *T*_s_ values (Table 1). This fact, as mentioned before, is associated with the cross-linking density of the vitrimers, which increased with a higher content of lignin [PDV-SKL (1:1, w/w) and (1:0.8, w/w)]. Overall, these results indicate a good thermal stability comparable to that of other lignin-based thermosets such as lignin-derived polyesters with a *T*_s_ of 151 °C.^48^
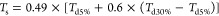
1

**Figure 3:**
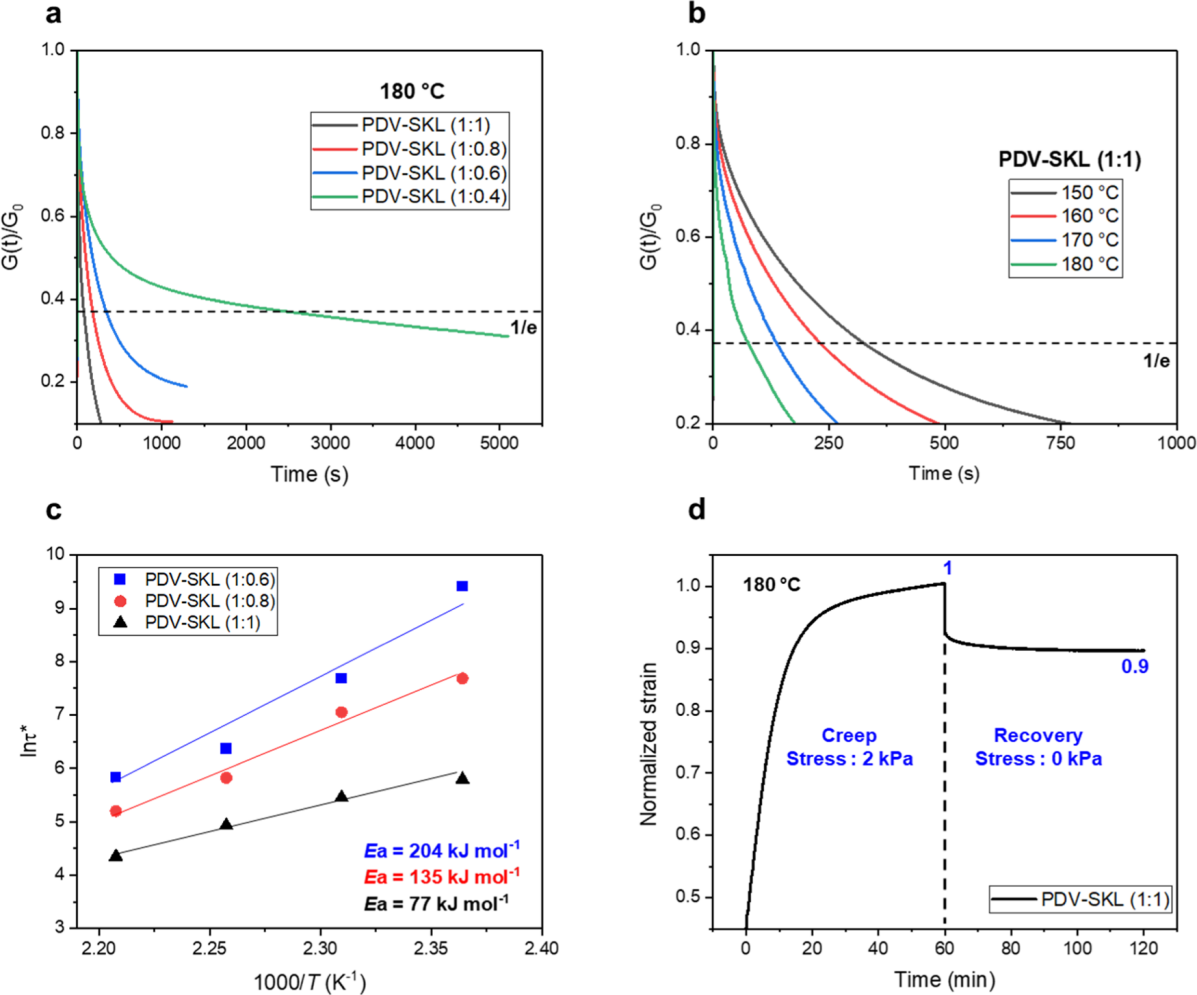
Dynamic mechanical characterization of lignin-based vitrimers (PDV-SKL): (a) stress relaxation curves of vitrimers with different lignin contents. (b) Stress relaxation curves of PDV-SKL (1:1) at different temperatures. (c) Fitting of the experimental relaxation times to the Arrhenius equation. (d) Creep-recovery behavior of PVD-SKL (1:1) at 180 °C.

The dynamic properties of lignin-based vitrimers were investigated by stress relaxation and creep studies using DMA. Figure 3a depicts the time-dependent relaxation time for all the lignin-based vitrimers at 180 °C (which is higher than their *T*_g_ values). All four compositions exhibited obvious stress relaxation curves at 180 °C, indicating their vitrimer behavior. Based on the Maxwell model for viscoelastic fluids, relaxation time (τ*) is defined as the time when the modulus of the sample decreases to 1/*e* with respect to the initial modulus. In this sense, the sample with the highest content of lignin [PDV-SKL (1:1)] exhibited the fastest relaxation rate with a relaxation time of 77 s. Thereafter, decreasing the content of lignin resulted in an obvious decrease in the relaxation rate, as reflected by PDV-SKL (1:0.8) with a τ* of 181 s, PDV-SKL (1:0.6) with a τ* of 342 s, and PDV-SKL (1:0.6) with a τ* of 2500 s (Figure 3a). These results are explained by the fact that a higher lignin content led to an increased concentration of unreacted hydroxyl groups in the cross-linked matrix, which in turn promoted more favorable and faster acetal exchange reactions *via* the transacetalization mechanism. In order to study more in detail the viscoelastic properties of the lignin-based vitrimers, PDV-SKL vitrimers with lignin contents from 50 to 37 wt %, i.e., PDV-SKL (1:1), PDV-SKL (1:0.8), and PDV-SKL (1:0.6), were selected, and stress relaxation curves at different temperatures were recorded. As could be expected from their thermal properties discussed above, the stress relaxation curve of PDV-SKL (1:1) achieved an absolute relaxation at the highest temperature (180 °C). An increase in the relaxation time was observed when decreasing the temperature, giving a temperature-dependent behavior (Figure 3b). Similar stress relaxation behavior was observed also from the compositions with lower lignin contents (Figure S6). These relaxation trends follow the Maxwell model and can be fitted to the Arrhenius law using experimental relaxation times (τ*) (Figure 3c). Subsequently, the relation between ln(τ*) and 1000/*T* (eq 2) was used to obtain the activation energy. The activation energy (*E*_a_) decreased from 204 to 77 kJ mol^–1^ as the lignin content increased from 37 to 50%. This trend indicates that as the concentration of SKL increases, PDV-SKL vitrimers exhibit much faster relaxation rates (Figure 3a) and much lower equilibrium moduli, indicating better dynamic properties. This fact is directly related to the increased presence of dynamic acetal bonds in the samples with a higher content of SKL, which would promote the exchangeability of covalent acetal bonds *via* the acetal metathesis pathway. Additionally, as stated above, residual hydroxyl groups (aliphatic + phenolic) would also promote faster acetal exchange reactions *via* transacetalization. This is in good agreement with related systems in the literature such as acetal-based polystyrene vitrimers developed by Li and co-workers, with reported activation energies ranging from 100 to 140 kJ mol^–1^.^24^ Remarkably, the stress relaxation curve at lower temperature (150 °C) for the PDV-SKL (1:0.4) vitrimer shows a clear decrease in the stress relaxation behavior (Figure S7). This clear deviation from the vitrimer behavior in contrast to PDV-SKL vitrimers with a higher SKL content (compare Figure 3b with Figure S7) points out that the topological rearrangement of the dynamic covalent networks and ultimately the reprocessability of the material with a lower SKL content would be restricted to higher temperatures (180 °C).

Creep recovery behavior experiments were also conducted to prove the malleability of the PDV-SKL vitrimers. As shown in Figure 3d, PDV-SKL (1:1) flowed after an initial elastic response at 180 °C, and once the stress was revoked (after 60 min), only a slight decrease in the strain was detected, with 90% of elongation retained over time. Similar behavior was also observed for PDV-SKL (1:0.8) (Figure S8). This significant viscoelastic response is attributed to the acetal exchange reactions that occur at elevated temperatures, which allows topological rearrangements of the cross-linked polymeric matrix under stress. Additionally, thermodilatometry experiments using DMA with a controlled force were performed to determine the malleability temperature (*T*_mall_), which is the point at which the strain increases abruptly in response to the increasing temperature (Figure S9). Thus, the *T*_mall_ was 135 and 105 °C for PDV-SKL (1:1) and PDV-SKL (1:0.8), respectively. This behavior is associated to the accelerated acetal exchange reactions at elevated temperatures and proves the malleability of the materials as a consequence of the topological network rearrangements as mentioned before.
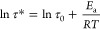
2

**Figure 4:**
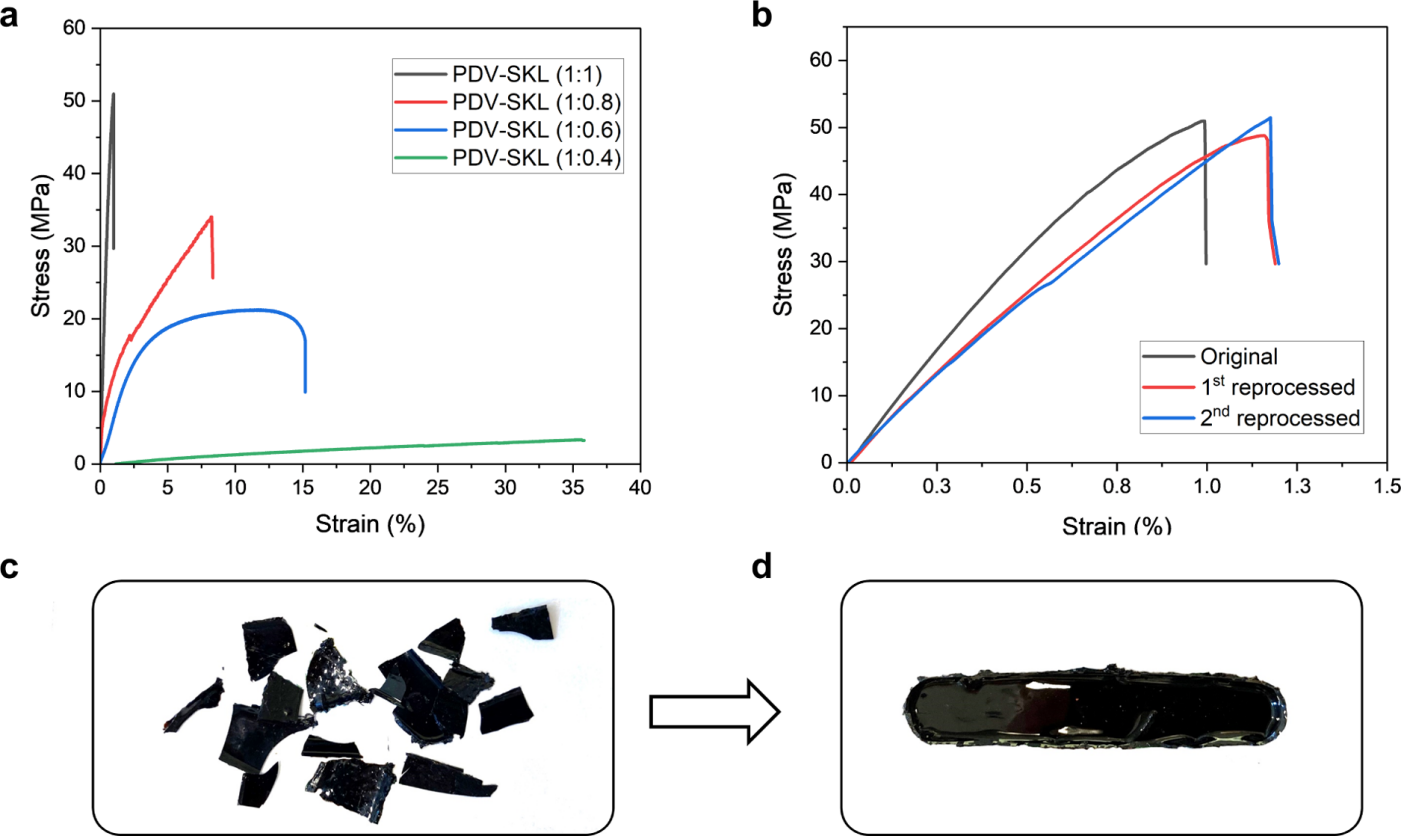
Mechanical characterization of lignin-based vitrimers (PDV-SKL): (a) stress–strain curves for PDV-SKL vitrimers with different SKL contents. (b) Stress–strain curves for PDV-SKL (1:1) vitrimer for different recycling cycles. (c) Digital image of a broken and cut PDV-SKL (1:1) vitrimer sample. (d) Digital image of a reprocessed PDV-SKL (1:1) vitrimer.

Mechanical properties of vitrimers largely define their suitability to different applications. The mechanical properties of all PDV-SKL vitrimers were examined by uniaxial tensile testing after equilibrating the specimens at 25 °C and 50% relative humidity for 24 h. Results are summarized in Figure 4a and Table 1. The introduction of flexible poly(ethylene) segments originating from PDV rendered the PDV-SKL vitrimers with tunable mechanical properties by simply adjusting the mass fraction of PDV in the reaction mixture (Figure S10).^48^ In this sense, the PDV-SKL (1:1) vitrimer behaved like a rigid plastic with only 1.0% elongation at break and a tensile strength of 50.9 MPa, which is significantly higher than that reported previously for other lignin-based vitrimers (12.9 MPa),^13^ and also lignin nanoparticle-reinforced polystyrene composites at 1.5% elongation at break.^47^ In the case of the PDV-SKL (1:0.8) vitrimer, an increase in the elongation at break (reaching 8.1%) was associated with a lower tensile strength (33.9 MPa), indicating a tougher material, while in the case of the PDV-SKL (1:0.6) vitrimer, these changes were more pronounced with 15% elongation at break and a tensile strength of 15.1 MPa. Lastly, the PDV-SKL (1:0.4) vitrimer exhibited significantly increased elastomeric behavior in comparison to the other compositions with 35% elongation at break. As discussed above, increasing the lignin content results in increased cross-linking density and rigidity of the vitrimer, which translates to an increase in the tensile strength and a decrease in the elongation at break. Overall, these results demonstrate unequivocally that the mechanical properties of PDV-SKL vitrimers can be modified by tuning the mass fraction of lignin and envision the possibility to produce specific lignin-based vitrimers with predictable mechanical properties as deduced from the linear correlation between the tensile strength (*R*^2^ = 0.98) or elongation at break (*R*^2^ = 0.87) with the lignin content (Figure S11).

Reprocessability is a key factor to evaluate the potential of vitrimer materials for circular applications and was here assessed using tensile testing. For this purpose, the mechanically failed vitrimer specimen PDV-SKL (1:1) was taken as a representative sample and reformed *via* compression molding at 150 °C for 2 h (Figure 4c,d). Tensile testing and reprocessing were repeated two times, and the stress–strain curves were successively recorded (Figure 4b). Results showed that the mechanical properties of the reprocessed PDV-SKL (1:1) vitrimer remained essentially unchanged, with the tensile strength ranging from 50.9 to 48.1 MPa with little variation in the elongation at break ranging from 1.0 to 1.2% (Table S2). No signs of degradation were found in the FT-IR spectra and DSC thermograms of the recycled vitrimers (Figures S12 and S13). Taken together, these data demonstrate mechanical and chemical stability of the PDV-SKL vitrimers as thermally processable circular materials.

**Figure 5:**
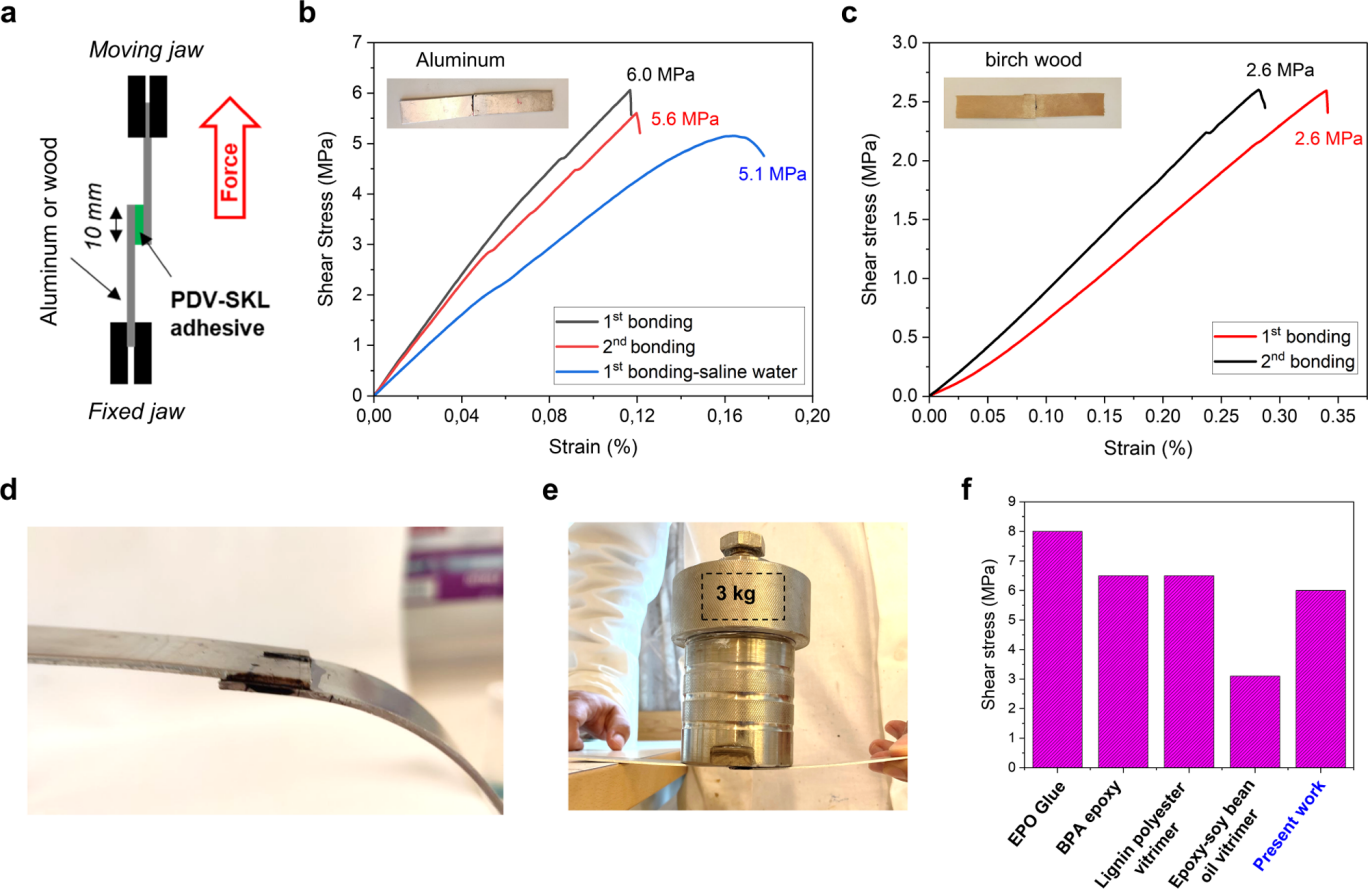
Application of PDV-SKL vitrimers as recoverable adhesives for aluminum and wood: (a) schematic representation of the lap shear test used in this work. (b) Lap shear test of PDV-SKL (1:1) as an adhesive on aluminum sheets with different bonding times. (c) Lap shear test of PDV-SKL (1:1) as an adhesive on birch wood sheets with different bonding times. (d,e) Digital images of PDV-SKL (1:1) as an adhesive on aluminum sheets during bending or holding an iron reactor. (f) Comparison between the adhesion performance of the PDV-SKL (1:1) vitrimer and related previously reported systems in the literature.

After confirming their ability to be reprocessed, the potential application of the lignin vitrimers as a recoverable adhesive was investigated. Again, the PDV-SKL (1:1) vitrimer was selected as a representative sample owing to its high lignin content and the highest tensile strength. First, aluminum was selected as a substrate for the adhesion test since it is the most consumed nonferrous metal.^62^ Adhesion samples were prepared by bonding two aluminum specimens with precured PDV-SKL (1:1). After the main curing stage at 150 °C, the two aluminum sheets bonded together exhibited a lap shear strength of 6.0 MPa (Figure 5a,b). Here, it is important to note that the level of adhesion strength stands comparison to that of commercial adhesives such as epoxy-amino glue (8 MPa),^63^ bisphenol A based epoxides (6 MPa),^64^ and other lignin-based vitrimers (6.5 MPa)^13^ and even outperforms biobased epoxy-vitrimers from soy bean oil (3.4 MPa)^38^ (Figure 5f). As demonstrated above, the PDV-SKL vitrimers have the potential to be thermally reprocessed because of the topological rearrangement enabled by the catalyst-free acetal exchange reactions.^24^ Therefore, the two specimens recovered after the adhesive strength tests were easily glued again *via* hot pressing at 150 °C for 2 h and subsequently exhibited a lap shear test of 5.6 MPa (Figure 5b). This result indicates preservation of the adhesion performance by 93%, significantly higher than that reported for other lignin-based vitrimers (76%)^13^ or other biobased vitrimers (85%).^38^ The adhesion performance was also evaluated under challenging conditions as was the immersion in highly concentrated saline water (32 wt %) during 100 h, with only a minor consequential decrease in the lap shear test (5.1 MPa), demonstrating promising applicability to environments such as marine or other challenging conditions (Figure 5b). Here, it is worth mentioning that the adhesive strength of PDV-SKL (1:1) was also demonstrated by bending the bonded aluminum plates (Figure 5d) or holding a 3 kg iron reactor directly at the site of adhesion (Figure 5e) without failure.

Last but not least, in order to explore the versatility of the PDV-SKL (1:1) vitrimer as an adhesive, nontreated birch wood was also tested owing to the broad use of sawn wood furniture items and construction materials. In this case, the lap shear test was measured at 2.6 MPa, which also stands comparison to that of other lignin-epoxy resins (2.0–2.4 MPa) used for the same purpose but without the possibility to be reprocessed (Figure 5c).^65^ In our case, the samples showed identical results in the lap shear test, which translates to 100% preservation of the adhesion performance, which is promising for the introduction of the lignin-based vitrimers to technological applications (Figure 5c). Here, it is important to note that in both cases (aluminum and wood), the adhesive was found on both glued specimens after the mechanical test (Figure S14), which indicates that the lap shear damage was caused by cohesive failure, not by adhesive failure between the substrate (aluminum or wood) and the adhesive [PDV-SKL (1:1) vitrimer].

## Conclusions

We have reported the preparation of lignin-based vitrimers with widely tunable mechanical properties and an excellent ability to be reprocessed without detrimental side effects. The salient features of our approach are the experimental ease, since there is no need for prior chemical modification of lignin, and the ability to conduct the cross-linking reaction without catalysts, thus offering a readily scalable route to lignin-based vitrimers in a simple way. The potential of the resulting lignin-based vitrimers was also proved by their application as recoverable adhesives for aluminum and wood, suggesting their broad potential in gluing hard and soft substrates. The lignin vitrimer adhesives were comparable to benchmark materials but displayed markedly higher preservation of adhesion strength for reglued samples. Taken together, our results encourage further work to explore potential industrial applications in circular adhesive materials. Finally, we also hold a view that our straightforward synthetic methodology will open up new avenues for the application of lignin-based vitrimers for advanced materials in versatile applications.

## Experimental Section

### Materials

All lignin-based vitrimers prepared in this work were prepared from BioPiva 100 pine kraft lignin (SKL) (UPM, Finland), previously characterized.^50^ Poly(ethylene glycol) divinyl ether (PDV, *M*_n_ = 250 g/mol) and 1,4-dioxane (99.9%), isopropanol, tetrahydrofuran, acetic acid (99%), guaiacol, and butyl vinyl ether were purchased from Sigma-Aldrich and used as received. Aluminum sheets (type 6061) and nontreated birch wood sheets were used as substrates for the lap shear test.

### Small-Molecule Model Experiments

#### Synthesis of 1-(1-butoxyethoxy)-2-methoxybenzene (AG) and Effect of Carboxylic Acid in the Formation of Acetal Bonds

To synthesize 1-(1-butoxyethoxy)-2-methoxybenzene (acetal guaiacol, AG), guaiacol (50 mmol) and butyl vinyl ether (75 mmol) were mixed without a solvent in the presence or absence of acetic acid (4.27 mmol) and stirred at 110 °C during 6 h. Conversion of guaiacol was monitored by ^1^H NMR spectroscopy at different times using the ratio of the acetal signal from AG (5.4 ppm)/free phenolic groups of guaiacol (5.65 ppm). The final product (AG) was purified by complete evaporation under reduced pressure and heat to remove the excess of butyl vinyl ether and extraction with dichloromethane/water (pH = 10) to remove the remaining free guaiacol and to obtain AG as a colorless liquid in 85% yield.

#### Catalyst-Free Dynamic Acetal Exchange Reactions

AG (1 mmol) and isopropanol (0.5 mmol) were mixed and stirred at 80 °C during 5 h. After that, the reaction mixture was concentrated to remove remaining isopropanol and analyzed by ^1^H NMR spectroscopy.

### Preparation of Lignin-Based Vitrimers

This procedure is representative of all the lignin-based vitrimers produced herein. The preparation of PDV-SKL (1:1) is described as a representative example. 1 g of lignin (SKL) was added to a vial with a magnetic stirrer, together with 3 mL of dioxane and 1 g of PDV. The reaction mixture was stirred at room temperature for 3 h to ensure complete dissolution of lignin. After that, the mixture was transferred to a round aluminum plate and placed in an oven at 110 °C during 6 h to evaporate dioxane and start the curing reaction. Next, the precured sample was placed in a Teflon mold with a rectangular shape and dimensions of 30 mm × 5 mm using a gauge length of 1 mm and cured at 150 °C for an additional 12 h.

### Characterization

#### Fourier Transform Infrared Spectroscopy

FT-IR was used to confirm that the cross-linking reaction between SKL and PDV had occurred. The measurements and spectra were recorded on a Varian 610-IR FT-IR spectrometer. The IR absorbance of the samples was measured in the range of 450–4000 cm^–1^ with attenuated total reflection–Fourier transform infrared spectroscopy (ATR-FTIR).

#### Gel Content

The pre-weighed lignin-based vitrimers (0.5 g) were extracted with a solvent mixture of tetrahydrofuran/dioxane (3:1, v/v) (150 mL) for 48 h at 70 °C. The insoluble fraction was then vacuum-dried at 50 °C until a constant weight was reached. The gel content was calculated according to the following equation
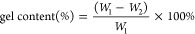


*W*_1_ and *W*_2_ represent the sample dry mass before and after the extraction, respectively.

#### ^31^P NMR Spectroscopy

The content of hydroxyl groups in SKL was previously determined by ^31^P NMR spectroscopy.^53^ Details of the method used are reported in a standard procedure.^66^ Briefly, the dry lignin sample (30 mg) is phosphitylated with 2-chloro-4,4,5,5-tetramethyl-1,3,2-dioxaphospholane (TMDP) (0.9 mmol) in the presence of *N*-hydroxy-5-norbornene-2,3-dicarboxylic acid imine (NHND) (0.010 mmol) as an internal standard and chromium(III) acetylacetonate as a relaxation agent. The ^31^P NMR experiments (256 scans, 10 s relaxation delay) were performed with 90° pulse angle and inverse gated proton decoupling.

#### Differential Scanning Calorimetry

Differential scanning calorimetry (DSC) measurements of the different lignin-based vitrimers was performed on Netzsch DSC 214 Polyma to determine the glass transition temperatures (*T*_g_), taken as the midpoint of the transition, of the vitrimer samples. N_2_ was used as the purge gas (50 mL/min), with a heating rate of 10 °C/min in the 25–250 °C temperature range. Calibration was performed using an indium standard for heat flow calibration and zinc standard for temperature calibration.

#### Thermogravimetric Analysis

The thermal stability of the lignin-based vitrimers was evaluated by TGA using a Discovery TG instrument (TA Instruments, USA) under 50 mL min^–1^ nitrogen flow with a temperature range of 30–700 °C and at a heating rate of 10 °C min^–1^.

#### Dynamic Mechanical Thermal Analysis

Dynamic mechanical properties were measured using a dynamic mechanical analyzer (DMA850) in the tension mode. The sample with dimensions of 30 mm × 5 mm × 1 mm was scanned from −50 to 200 °C at a heating rate of 3 °C min^–1^. The amplitude was set at 15 μm, and the frequency was 1 Hz.

#### Stress Relaxation Test

Stress relaxation was measured using a dynamic mechanical analyzer (DMA850). The sample dimension was 30 mm × 5 mm × 1 mm. The sample was heated to the target temperature and thermally equilibrated for 15 min with a static force of 0.01 N. After that, a strain of 5% was applied to the sample, and the stress and modulus were recorded over time until reaching equilibrium.

#### Creep Recovery Test

Creep recovery behavior was measured using a dynamic mechanical analyzer (DMA850). The sample dimension was 30 mm × 5 mm × 1 mm. First, the sample was heated at 180 °C and for 5 min; then, a constant force was applied (2 kPa) to the material for 1 h. After that, the force was revoked and the material recovered for an additional hour.

#### Thermodilatometry Test

Thermodilatometry was measured using a dynamic mechanical analyzer (DMA850) in the controlled-force tension mode. The sample dimension was 30 mm × 5 mm × 1 mm. A constant force (2 kPa) was applied to stretch the material and made the stress constant, and the sample was heated to 250 °C at a heating rate of 5 °C min^–1^.

#### Tensile Properties

The mechanical properties of the lignin-based vitrimers were measured using an Instron 5960 universal testing machine (Instron, USA) equipped with a 100 N load cell at a strain rate of 1 mm min^–1^. The mechanical measurements were performed on rectangular-shaped specimens with dimensions of 30 mm × 5 mm using a gauge length of 1 mm. The specimens were conditioned 24 h prior to the measurement and measured at 50% relative humidity (RH) and 25 °C. Young’s modulus was calculated from the slope of the linear part of the stress–strain curve. The results are reported as mean values ± standard deviation of a minimum of three samples.

#### Lap Shear Test

The adhesion performance of the lignin-based vitrimers was evaluated using an Instron 5960 universal testing machine (Instron, USA) equipped with a 10 kN load cell at a strain rate of 5 mm min^–1^ according to the ISO 4587:2003. Aluminum sheets (6061) with a dimension of 100 mm × 25 mm × 1 mm were polished using sand paper and then cleaned using distilled water and 2-propanol successively. Nontreated birch wood sheets with a dimension of 60 mm × 15 mm × 1 mm were used without further treatment. The pre-cured PDV-SKL (1:1) vitrimer (obtained as described in the Preparation of Lignin-Based Vitrimers) was applied on the wood and aluminum samples (adhesive loading of 208 g m^–2^) with an overlapping area of 12.5 mm × 25 mm and 15 mm × 7.5 mm for aluminum and wood, respectively. After that, the samples were sandwiched, pressed (1 kN), and then cured at 150 °C for 3 h. At least four repetition tests were performed for each sample.

## Supporting Information Available

31P NMR of SKL and quantification of hydroxyl groups; DSC of “click” curing process between SKL and PDV; stress relaxation curves for PDV-SKL vitrimers at different temperatures; creep recovery test for the PDV-SKL (1:0.8) vitrimer; thermodilatometry experiments for PDV-SKL vitrimers (1:1) and (1:0.8); digital images of PDV-SKL vitrimers; FTIR-spectra and DSC thermograms of the PDV-SKL (1:1) vitrimer before and after their reprocessability; and digital images of aluminum and wood sheets after the adhesion test (PDF)

## Notes

^#^ A.M. and M.M. contributed equally to this work.
