# Supplementary material for: Catalyst-Free Synthesis of Lignin Vitrimers with Tunable Mechanical Properties: Circular Polymers and Recoverable Adhesives
Source: ACS Appl Mater Interfaces. 2021 Nov 23;13(48):57952–61. doi: 10.1021/acsami.1c17412 (PMC8662642; doi:10.1021/acsami.1c17412)
Supplement: Supplementary file 1 — am1c17412_si_001.pdf [file am1c17412_si_001.pdf]

Supporting Information for

Catalyst-Free Synthesis of Lignin Vitrimers with Tunable Mechanical Properties: Circular Polymers and Recoverable Adhesives

Adrian Moreno,*[a]† Mohammad Morsali, [a]† Mika H. Sipponen*[a]

†*These authors contributed equally to this work.*

[a] *Department of Materials and Environmental Chemistry, Stockholm University, 10691, Stockholm (Sweden)*

**Corresponding authors:*
adrian.morenoguerra@mmk.su.se
mika.sipponen@mmk.su.se

This supporting information contains:

Total Number of Pages: 12

Total Number of Figures: 14

Figure S1 shows the model reactions employed to elucidate the effect of the presence of carboxylic acid in the thermal induced click reaction between guaiacol and butyl vinyl ether. Acetic acid was used as model carboxylic acid, in a concentration of 0.057 mol respect the phenolic group guaiacol to mimic the same composition present in SKL.

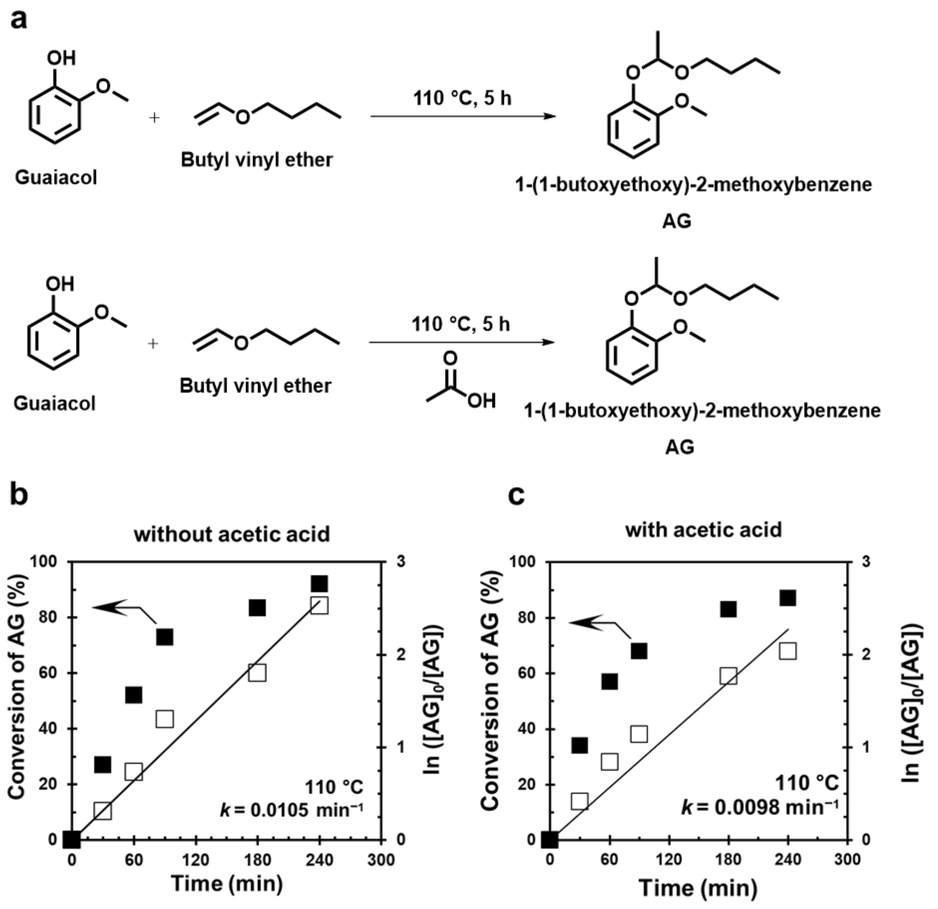

Figure S1. (a) Model reactions employed to elucidate the effect of carboxylic acid during acetal formation via kinetic experiments. Kinetics experiments from the reaction between guaiacol and butyl vinyl ether (b) without acetic acid and (c) in the presence of acetic acid.

Figure S2 shows the reactions employed to elucidate the mechanism behind the dynamic exchange of acetal bonds. The derived coupling product between guaiacol and butyl vinyl ether, 1-(1-butoxyethoxy)-2-methoxybenzene (AG) was used as a model product and reacted with isopropanol (IPA) to simulate the dynamic exchange reactions of the vitrimers. The reaction mixture after 5 h at 80 °C confirm the formation of the corresponding transacetalization products from the formation of the new acetal signals (2 and 3) from the initial acetal signal 1 corresponding to AG, according to ^{1}H NMR spectroscopy (Figure S2). Products derived from the acetal metathesis between the initial acetal (AG) and the derived from transacetalization were also detected trough the formation of new acetal signals (a, b and c) (Figure S2). These results indicate that the combination of transacetalization and acetal metathesis is responsible for the catalyst-free dynamic acetal exchange reactions.

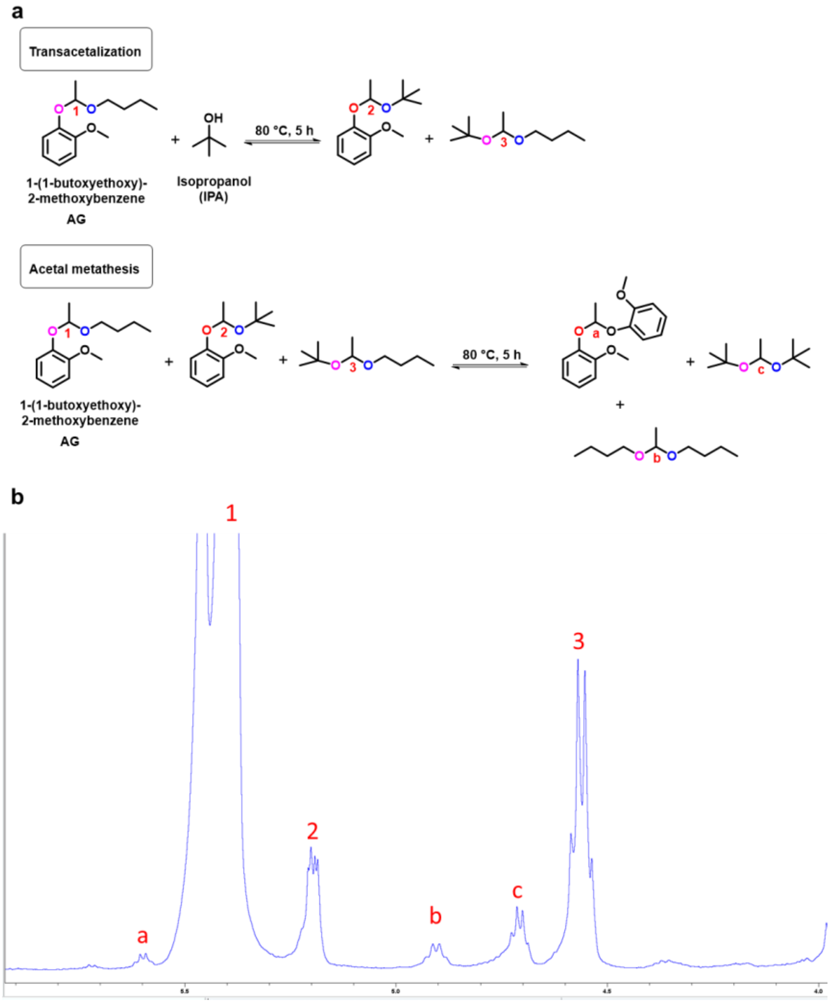

Figure S2. (a) Catalyst-free acetal exchange reactions via transacetalization and acetal metathesis. (b) ^{1}H NMR spectra of the small model compounds reaction mixture after 5 hours at 80 °C in $CDCl_3$.

Figure S3 shows the ^{31}P NMR spectra of the original SKL employed for the preparation of lignin-based vitrimers.

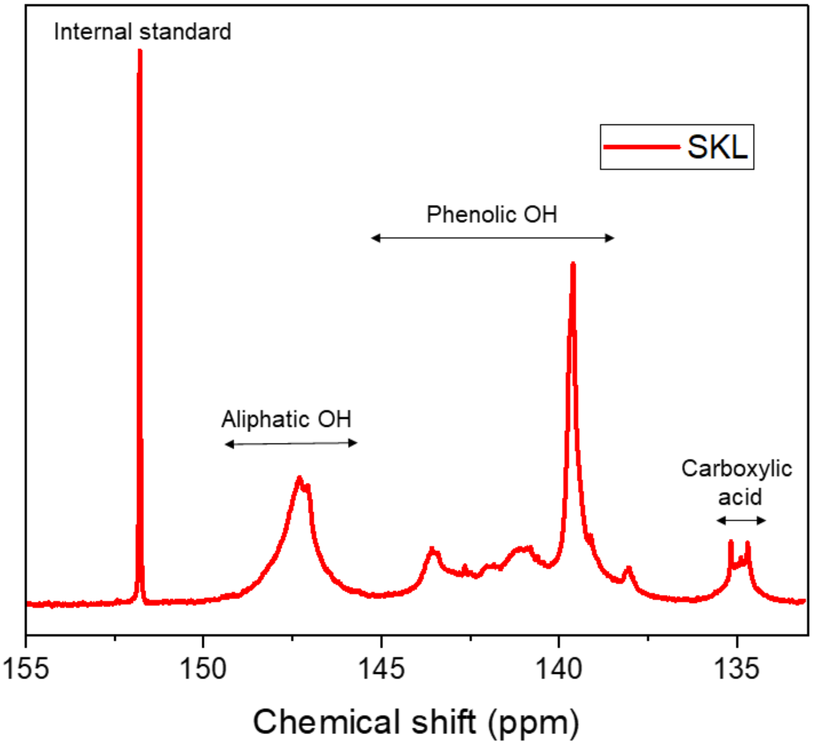

Figure S3. Quantitative ^{31}P NMR spectra of SKL.

Table S1 shows the quantitative analysis of hydroxyl groups in SKL used for the preparation of lignin-based vitrimers (PDV-SKL).

Table S1. Concentration of Aliphatic, Phenolic and carboxylic OH of SKL according to quantitative ^{31}P NMR.[a]

sample	aliphatic OH	phenolic OH	Carboxylic acid OH	Total OH
SKL	1.89 ± 0.04	4.05 ± 0.01	0.36 ± 0.01	6.30 ± 0.09

[a] Unit mmol/g. At least three measurements were completed for each parameter. Error ranges correspond to one standard deviation. Data is from Moreno, A.; Liu, J.; Gueret, R.; Hadi, E. S.; Bergström, L.; Slabon, A.; Sipponen, M. H. Unravelling the Hydration Barrier of Lignin Oleate Nanoparticles for Acid- and Base-Catalyzed Functionalization in Dispersion State. Angew. Chem. Int. Ed., 2021, 60, 20897-20905.

Figure S4 shows that signal associated to the C=C stretching bond at 1460 cm^{-1} completely disappear after the thermal "click" addition (red dashed section). This fact, is also supported by the unequivocally absence of the of the stretching band corresponding to the double bond (=C-H, 3005 cm^{-1}) as stated in the manuscript. The signal around 1600 cm^{-1} present PDV-SKL vitrimer and also in pristine Kraft lignin (SKL) with the same intensity, (blue dashed section) is associated to the aromatic skeleton vibration of the inherent structure of lignin.

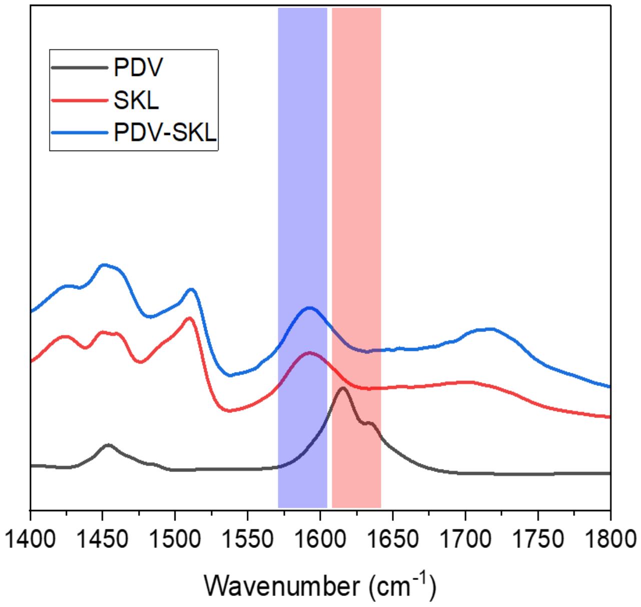

Figure S4. Magnification of FT-IR spectra of SKL, PDV and PDV-SKL (1:1).

Figure S5 shows the non-isothermal peak corresponding to the thermal-induced "click" reaction of PDV with SKL.

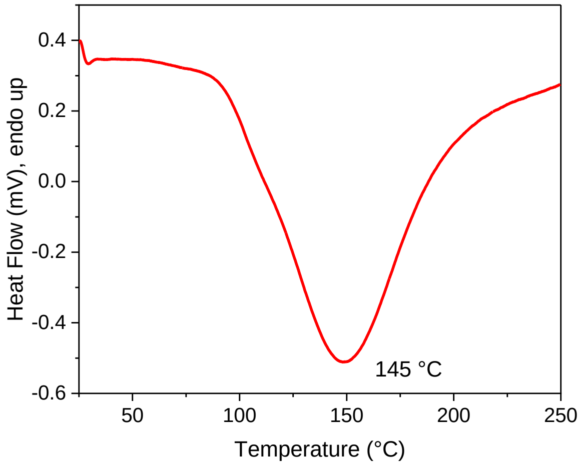

Figure S5. DSC curve of the "click" reaction between SKL and PDV to form PDV-SKL (1:1)

Figure S6 shows the time-dependence relation between the relaxation time for PDV-SKL vitrimers at different temperatures.

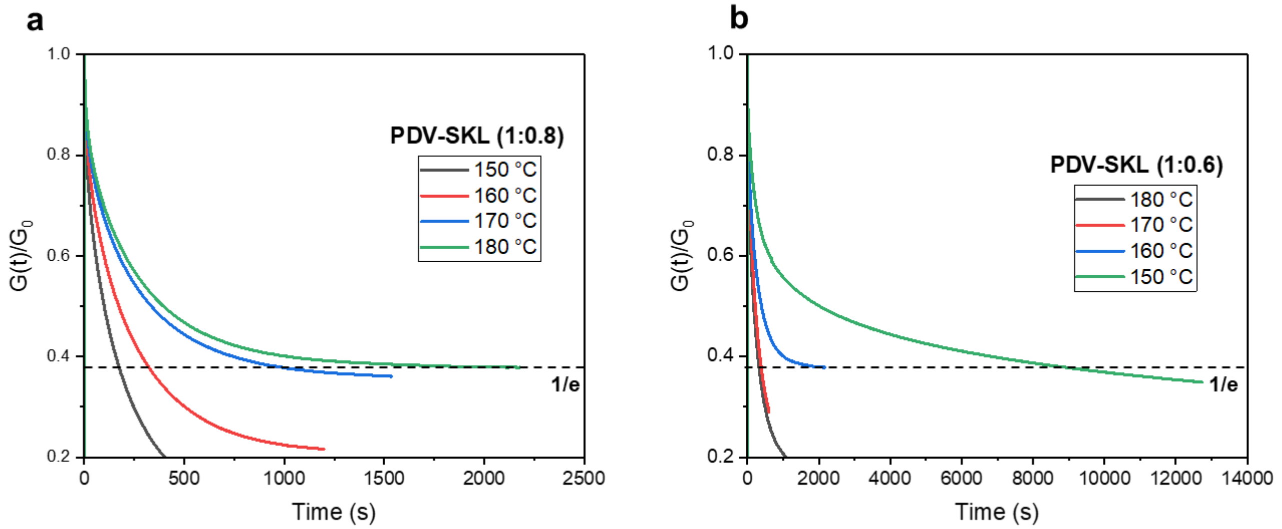

Figure S6. Stress-relaxation curves of (a) PDV-SKL (1:0.8) and (b) PDV-SKL (1:0.6) at different temperatures.

Figure S7 shows the clear decrease on the stress-relaxation curve for PDV-SKL vitrimer (1:0.4) at 150 °C.

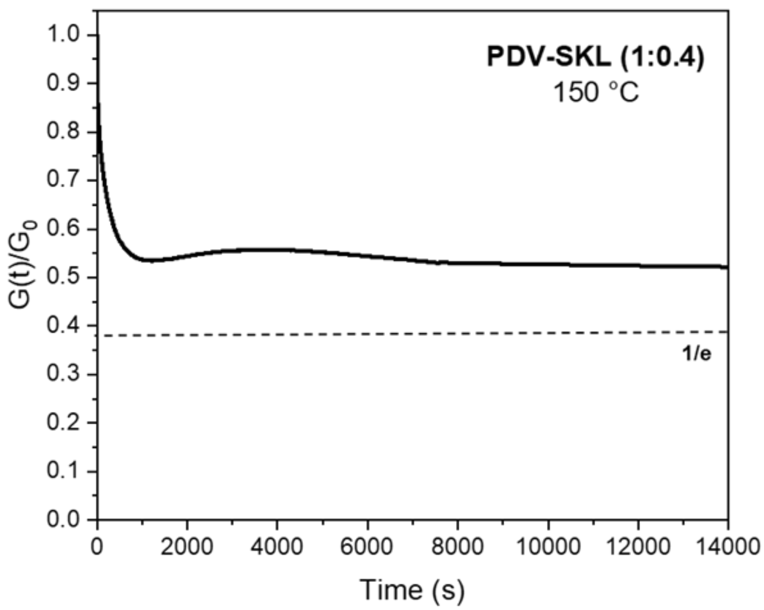

Figure S7. Stress relaxation curve of PDV-SKL (1:0.4) at 150 °C.

Figure S8 shows the clear viscoelastic response of PDV-SKL (1:0.8) during creep-recovery test.

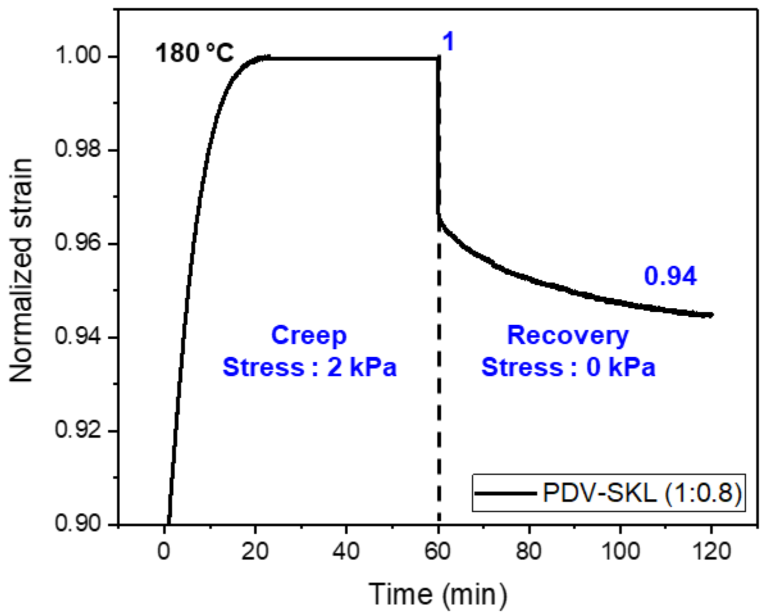

Figure S8. Creep-recovery behaviour of PVD-SKL (1:0.8) at 180 °C.

Figure S9 shows the malleability temperature (T_{mall}) for PDV-SKL vitrimers, which is defined as a clear increase in the expansion coefficient.

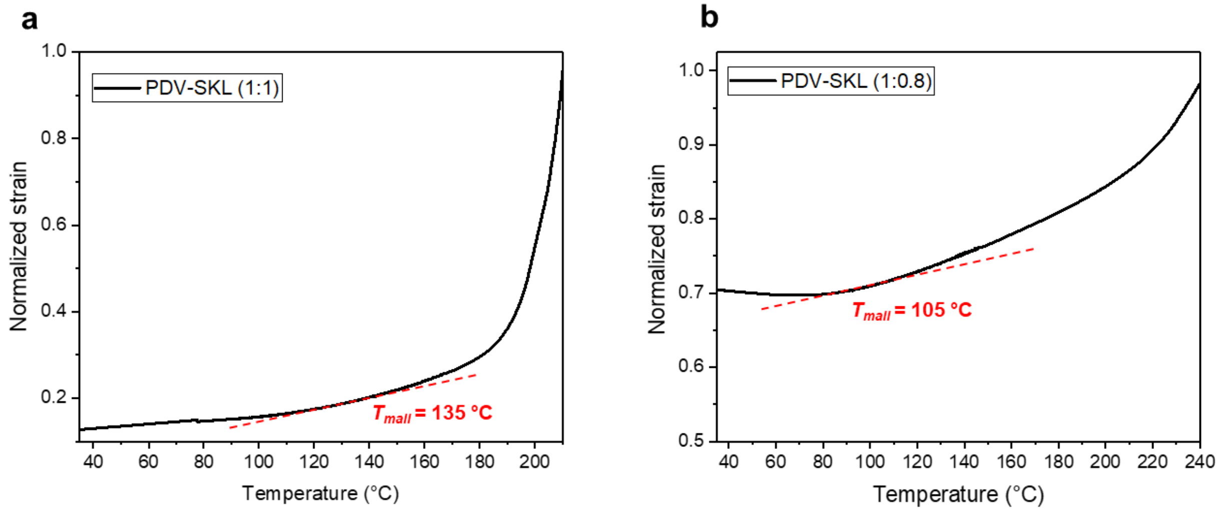

Figure S9. Curve for thermodilatometry of (a) PDV-SKL (1:1) and (b) PDV-SKL (1:0.8)

Figure S10 shows how the rigidity of the lignin-based vitrimers (PDV-SKL) is affected by decreasing the amount of lignin (SKL) in the system.

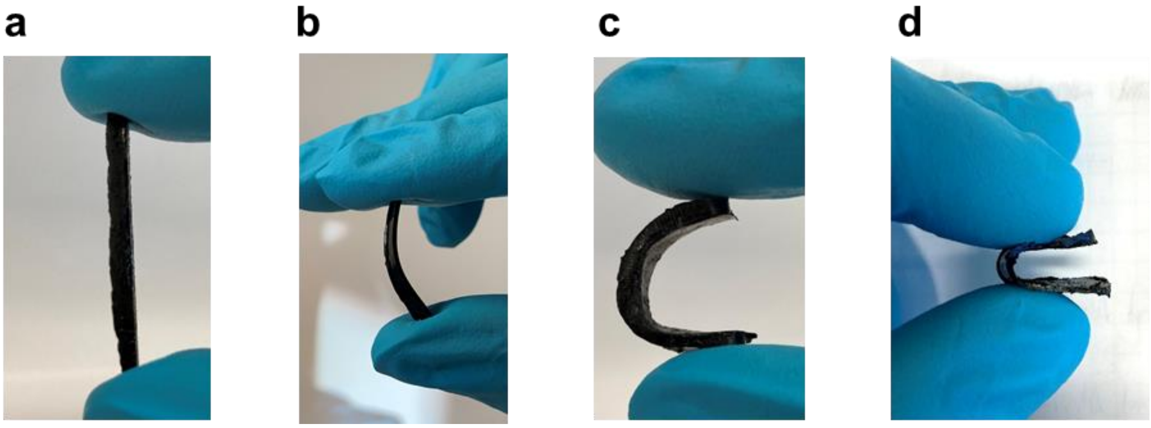

Figure S10. Bending ability of lignin-based vitrimers with different lignin content: (a) PDV-SKL (1:1), (b) PDV-SKL (1:0.8), (c) PDV-SKL (1:0.6) and (d) PDV-SKL (1:0.4).

Figure S11 shows the linear correlation between mechanical properties and SKL content for the different lignin-based vitrimers.

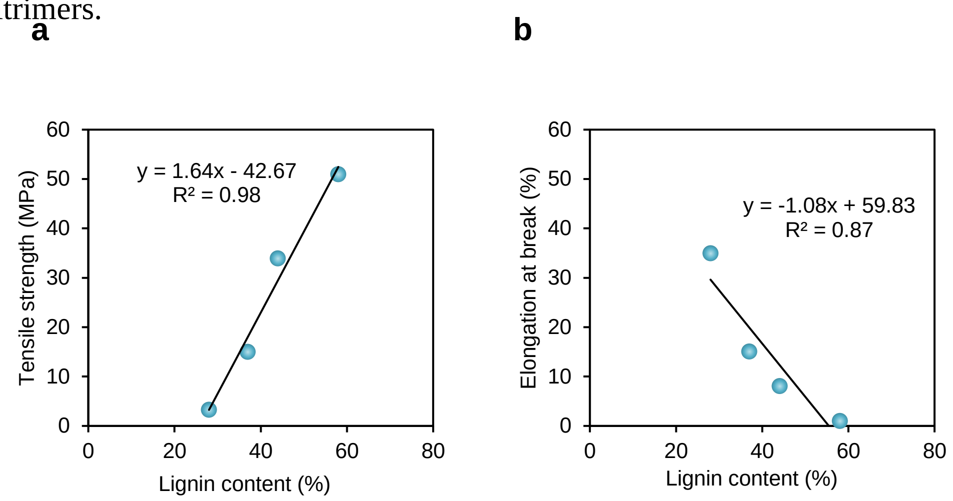

Figure S11. Correlation between (a) tensile strength (MPa) and (b) elongation at break (%) with lignin content (wt %).

Table S2 shows the mechanical properties of PDV-SKL (1:1) vitrimer before and after its reprocessability via compression molding at 150 °C for 2 hours.

Table S2. Composition of PDV-SKL (1:1) vitrimers and their thermal and mechanical properties.

Sample	Modulus (MPa)	Tensile strength (MPa)	Elongation (%)
PDV-SKL (1:1)	2100	50.9	1.0
PDV-SKL (1:1)-1st reprocessed	1870	50.1	1.27
PDV-SKL (1:1)-2nd reprocessed	1840	48.1	1.29

Figure S12 shows the FT-IR spectra for PDV-SKL (1:1) vitrimer before (black line), and after their reprocessability (blue and red line). The absence of any significant change in contrast to the original sample (black line) indicate a good reprocessability behavior, and the absence of degradation events.

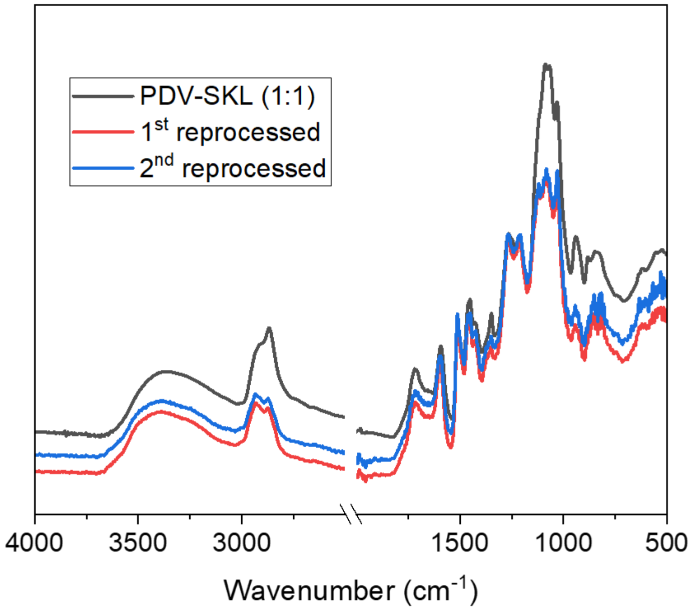

Figure S12. FTIR spectra of the original PDV-SKL (1:1) vitrimer (black) and reprocessed (blue and red).

Figure S13 shows the DSC thermograms for PDV-SKL (1:1) vitrimer before (black line), and after their reprocessability (blue and red line). The absence of any change in contrast to the original sample (black line) indicate a good reprocessability behavior, and the absence of degradation events.

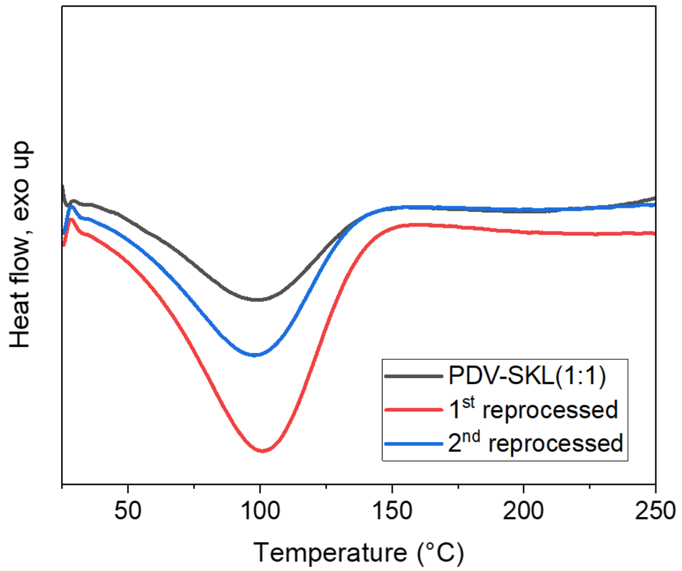

Figure S13. Non-isothermal DSC curves of the original PDV-SKL (1:1) vitrimer (black) and reprocessed (blue and red).

Figure S14 shows the presence of PDV-SKL (1:1) vitrimer adhesive in both sites of samples (aluminum and wood) after the adhesion test.

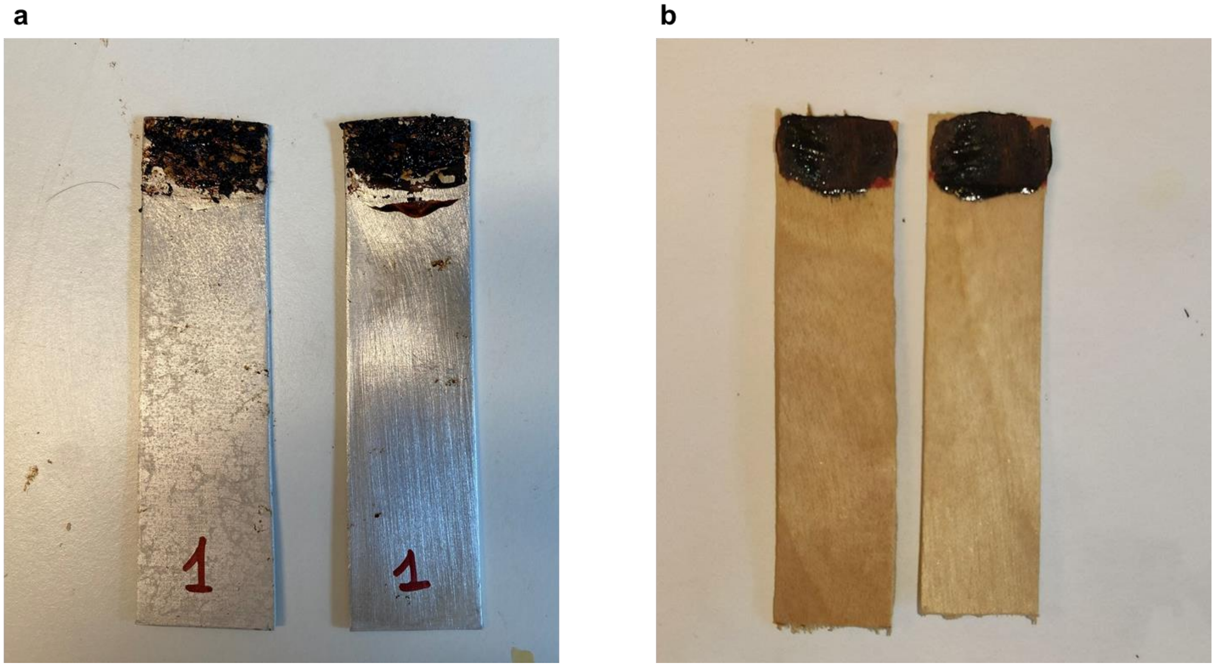

Figure S14. (a) Aluminium and (b) birch wood sheets after the adhesion test using PDV-SKL (1:1) vitrimer as recoverable adhesive.
